# Cell-type specific requirements for thiol/disulfide exchange during HIV-1 entry and infection

**DOI:** 10.1186/1742-4690-9-97

**Published:** 2012-12-03

**Authors:** Tzanko S Stantchev, Mark Paciga, Carla R Lankford, Franziska Schwartzkopff, Christopher C Broder, Kathleen A Clouse

**Affiliations:** 1 Laboratory of Cell Biology, Division of Monoclonal Antibodies, U.S. Food and Drug Administration, Bethesda, MD, 20892, USA; 2 Department of Microbiology and Immunology, F. Edward Hébert School of Medicine, Uniformed Services University, Bethesda, MD, 20814, USA

**Keywords:** HIV-1, Virus entry, Protein disulfide isomerase, Thioredoxin, Cell type specificity

## Abstract

**Background:**

The role of disulfide bond remodeling in HIV-1 infection is well described, but the process still remains incompletely characterized. At present, the data have been predominantly obtained using established cell lines and/or CXCR4-tropic laboratory-adapted virus strains. There is also ambiguity about which disulfide isomerases/ reductases play a major role in HIV-1 entry, as protein disulfide isomerase (PDI) and/or thioredoxin (Trx) have emerged as the two enzymes most often implicated in this process.

**Results:**

We have extended our previous findings and those of others by focusing on CCR5-using HIV-1 strains and their natural targets - primary human macrophages and CD4^+^ T lymphocytes. We found that the nonspecific thiol/disulfide exchange inhibitor, 5,5'-dithiobis(2-nitrobenzoic acid) (DTNB), significantly reduced HIV-1 entry and infection in cell lines, human monocyte-derived macrophages (MDM), and also phytohemagglutinin (PHA)-stimulated peripheral blood mononuclear cells (PBMC). Subsequent studies were performed using specific anti-PDI or Trx monoclonal antibodies (mAb) in HIV-1 envelope pseudotyped and wild type (wt) virus infection systems. Although human donor-to-donor variability was observed as expected, Trx appeared to play a greater role than PDI in HIV-1 infection of MDM. In contrast, PDI, but not Trx, was predominantly involved in HIV-1 entry and infection of the CD4^+^/CCR5^+^ T cell line, PM-1, and PHA-stimulated primary human T lymphocytes. Intriguingly, both PDI and Trx were present on the surface of MDM, PM-1 and PHA-stimulated CD4^+^ T cells. However, considerably lower levels of Trx were detected on freshly isolated CD4^+^ lymphocytes, compared to PHA-stimulated cells.

**Conclusions:**

Our findings clearly demonstrate the role of thiol/disulfide exchange in HIV-1 entry in primary T lymphocytes and MDM. They also establish a cell-type specificity regarding the involvement of particular disulfide isomerases/reductases in this process and may provide an explanation for differences among previously published studies. More importantly, from an *in vivo* perspective, the preferential utilization of PDI may be relevant to the HIV-1 entry and establishment of virus reservoirs in resting CD4^+^ cells, while the elevated levels of Trx reported in the chronic stages of HIV-1 infection may facilitate the virus entry in macrophages and help to sustain high viremia during the decline of T lymphocytes.

## Background

There have been an increasing number of studies supporting an important role for thiol (−SH)/disulfide (−S-S-) exchange in the entry of multiple viruses into susceptible cells, including Sindbis virus [1] , Baculovirus [2], Vaccinia virus [3], equine arteritis virus [4], Moloney murine leukemia virus [5], Newcastle disease virus [6-8], hepatitis delta virus [9] and HIV-1 (reviewed in [10,11]).

HIV-1 entry requires attachment of the gp120 subunit of the viral envelope (Env) glycoprotein to its primary receptor, CD4. This interaction induces a structural rearrangement in Env, exposing conserved regions within the gp120 subunit, thereby enabling binding to an appropriate co-receptor, primarily the chemokine receptors CXCR4 or CCR5 [12,13]. Engagement of gp120 to the co-receptor triggers conformational changes in the gp41 subunit, exposing its hydrophobic amino-terminal fusion peptide which inserts into the host cell membrane, followed by the formation of the six helix bundle (trimer-of-hairpin) structure that enables fusion of the virus and cell membranes and the subsequent release of the viral capsid into the cytosol (reviewed in [12,14].

HIV-1 Env contains 10 disulfide bonds (nine in the gp120 and one in the gp41 subunits, respectively) [15], and the observed potent suppression of HIV-1 infection by disulfide isomerase inhibitors has revealed the importance of thiol/disulfide exchange during virus entry [16-21]. At present, there is still ambiguity regarding which enzyme(s) mediate the disulfide bond rearrangements. The majority of studies investigating the effects of disulfide isomerase/reductase inhibitors [16-20,22], specific polyclonal and monoclonal antibodies (Ab) directed against these enzymes [16,18,20], as well as fluorescent microscopy and Western blot analysis of gp120 interactions and/or reduction of disulfide bonds [20,23,24], imply that PDI is the key enzyme involved in the HIV-1 entry process. However, it has also been reported [25] that Trx plays a major role during HIV-1 Env induced fusion, while PDI expression and/or function has a negligible effect. In yet another study [26], it was shown that another member of the Trx superfamily, glutaredoxin-1 (Grx1), can reduce disulfide bonds in both CD4 and gp120, and its inhibition results in reduced HIV-1 infection.

Thus, the significance of the thiol/disulfide exchange in the process of HIV-1 infection has been established predominantly using CD4/coreceptor-positive cell lines and laboratory-adapted, CXCR4-tropic (X4) HIV-1 strains, with a limited number of studies employing primary human cells and/or R5-tropic (R5) HIV-1 isolates [16,19,22,25,26]. R5 strains of HIV-1 are primarily involved in virus transmission; they predominate during the early and asymptomatic stages of infection, and are typically present throughout the symptomatic phase of HIV disease (reviewed in [27]). For these reasons, we conducted a comprehensive study using a panel of distinct assays to focus extensively on the significance of thiol/disulfide exchange during entry of several R5-tropic HIV-1 strains, including those that were minimally passaged, into their natural targets – primary human macrophages and CD4^+^ lymphocytes. In addition, we determined the effects of disulfide reductase/isomerase inhibitors on human macrophage infection with HIV-1_92UG024_, one of the few primary X4-tropic strains capable of infecting these cells [28]. Our results provide critical new information on the role of disulfide isomerases in HIV infection and further support previous observations that thiol/disulfide exchange is required for HIV-1 entry. More interestingly, our data reveal cell-type specificity with respect to the enzymes involved in this process.

## Results

### DTNB inhibits R5 Env pseudotyped virus infection in CD4^+^/CCR5^+^ cell lines, primary macrophages and PBMCs

**Figure 1:**
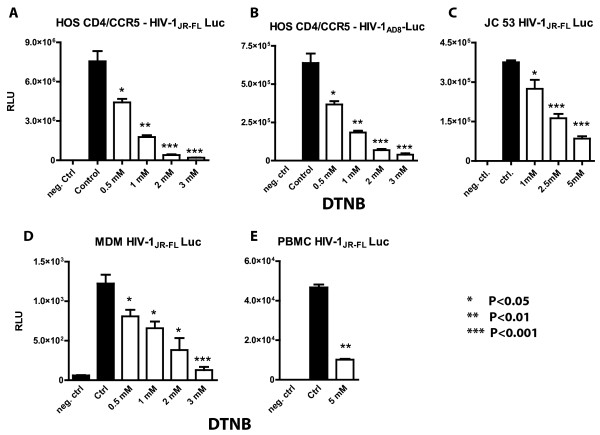
**The broad spectrum thio**-**isomerase inhibitor,DTNB,significantly reduces infection by HIV**-**1 Env pseudotyped virus particles.** The CD4^+^CCR5^+^ HOS (panel **A**,**B**) and JC53 (panel **C**) cell lines, as well as the primary human MDM (panel **D**) and PBMC (panel **E**) were pre-incubated with different concentrations of DTNB for 1 h and then infected with HIV-1-Env pseudotyped virions in the presence of the inhibitor. The infection was carried out for 2.5 h at 37°C in triplicate in a serum free medium. Cells were subsequently washed with DMEM and further incubated in DM-10 for 48 h prior to lysis (0.5% Triton-100 in PBS) and measurement of Luc activity. Cell lysates from mock-infected cells and from cells not treated with DTNB were used as a negative control and control, respectively. The P values for the differences between the control and DTNB treated cells were calculated using the GraphPad Prism scientific software (t-test).

We initially examined the effect of the nonspecific thiol/disulfide exchange inhibitor, DTNB, on the infection of different cell types by luciferase (Luc) reporter gene-encoding HIV-1_JR-FL_ or HIV-1_AD8_ Env pseudotyped virus particles. Since these particles were only competent for single cycle infection and were produced using the same backbone plasmid, their application allowed a more accurate assessment of the effect of DTNB on distinct HIV-1 Env-pseudotyped virions and/or experimental cell systems. After a brief pre-incubation (~1 h), cells were infected in the presence of DTNB for 2.5 h, followed by an extensive washout of the reagent. About 48 h later, cells were lysed, and Luc activity was measured and expressed in relative light units (RLU). In HOS CD4^+^/CCR5^+^ cells, DTNB exposure potently reduced infection by HIV-1_JR-FL_ or HIV-1_AD8_ Env-pseudotyped virions (Figure 1A) without impairing cell viability (trypan blue exclusion test). DTNB also caused a similar dose-dependent suppression of HIV-1_JR-FL_ Env-pseudotyped virion infection of the JC53 CD4^+^/CCR5^+^ cell line, as well as primary macrophages and PBMCs (Figure 1B,C,D), without significantly altering the cell surface expression of CD4 or chemokine co-receptors (data not shown). DTNB incubation also has no effect on Luc activity in cells directly electroporated with the pNL4-3 e-R+ Luc plasmid used to generate the reporter gene-encoding HIV-1 Env pseudotyped virus particles (data not shown), consistent with previous observations that DTNB is nearly plasma membrane impermeable [29,30].

### DTNB inhibits HIV-1 particle fusion with primary human cells and CD4^+^/CCR5^+^ cell lines

**Figure 2:**
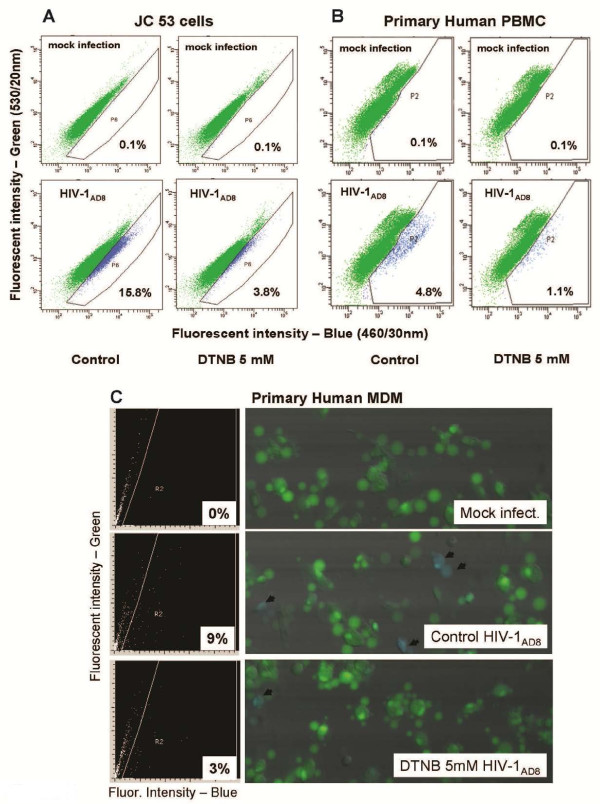
**DTNB inhibits fusion**/**entry of BlaM**-**containing virions in JC53 cells**, **primary human PBMCs and MDM.** The BlaM-containing virus particles were produced and the JC53 CD4+/CCR5+ cells, primary human MDM, and PBMCs were cultured as described in Methods. DTNB was dissolved in pre-warmed phenol red free DMEM, and cells were pre-incubated for 1 h before addition of the virus. The infection period was 2 h at 37°C in serum-free medium for the JC53 cells (panel **A**) and 2.5 h for the primary PBMCs (panel **B**) and MDM (panel **C**) in the presence of DTNB. After incubation with BlaM-containing virus particles, cells were washed with DMEM, loaded with the fluorescent dye CCF2/AM for 1 h at RT, washed and further incubated in DM-10 (phenol red free, supplemented with 2.5 mM Probenecid) for 14 h in tissue culture plates at RT in the dark. Subsequently, the JC53 cells (after trypsinization) and the human PBMCs were transferred to microfuge tubes, fixed with 1.6% pareformaldehyde and analyzed using BD LSR II Cell Fluorometer equipped with a violet laser (407 nm excitation wavelength) and 460/30 nm and 530/20 nm emission filters for detection of the blue and green fluorescence, respectively (**A**,**B**). The primary human MDM (**C**), cultured in a borosilicate glass bottom plate (Whatmann), were fixed *in situ* and analyzed using a Laser Scanning Cytometer (CompuCyte, Cambridge, MA). Mock-infected cells (upper panels) were used to define the regions of negative (green) and positive (blue) cell populations in the presented histograms. The experiment with human MDM was performed in triplicates. The infection of the JC53 cells and primary PBMC was carried out in duplicate wells, which were combined before Flow Cytometry analysis.

It is well-documented that the HIV-1 life cycle involves multiple steps that include viral Env-mediated membrane fusion, uncoating of viral RNA, reverse transcription, proviral DNA integration, transcription, and translation, as well as viral particle assembly and budding [31]. Given that DTNB could potentially interfere with multiple cell surface proteins containing sulphydryl groups, although unlikely, it was still possible that the effect of DTNB on HIV-1 replication could be nonspecific. For this reason, we sought to examine the effect of DTNB solely on virus entry by employing β-lactamase (BlaM)-containing virions [32,33] prior to testing its effect on spreading HIV-1 infection *in vitro*. Negative controls prepared for DTNB treated and untreated cells confirmed that DTNB had no detrimental effects on loading of the cells with the fluorescent dye, CCF2/AM, and did not influence the background non-specific cell fluorescence (Figure 2A, B, upper graphs). In addition, cells electroporated with pMM310 (pcDNA3.1(Zeo)-Vpr-BlaM), incubated in 5 mM DTNB and subsequently loaded with the fluorescent dye CCF2/AM showed no significant difference in the number of blue cells, compared to cells incubated in DMEM only (data not shown), indicating that DTNB did not affect the BlaM enzymatic activity. Using HIV-1_AD8_ BlaM containing virus particles, we established that DTNB significantly inhibited fusion of HIV-1_AD8_ BlaM-containing virions with both the JC53 CD4^+^/CCR5^+^ cell line (Figure 2A) and primary human PBMCs (Figure 2B), as well as MDMs (Figure 2C), indicating that its effect occurs predominantly at the level of virus entry and further supporting the role for thiol/disulfide exchange in HIV-1 Env during infection.

### Thiol/disulfide exchange is required for infection of human MDM by primary HIV-1 strains

**Figure 3:**
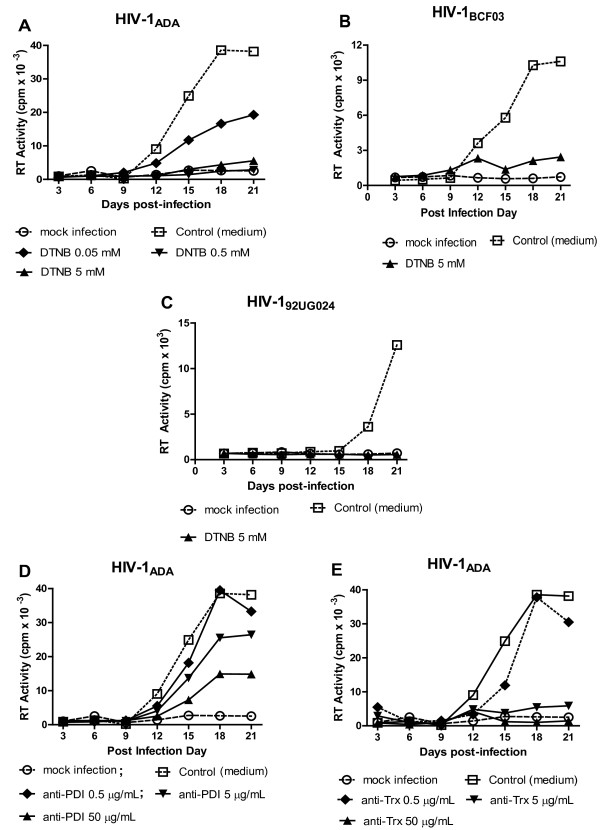
**Inhibition of thiol**-**disulfide exchange suppresses HIV**-**1 infection of primary human MDM.** After preincubation for 30 minutes. with various concentrations of DTNB, anti-PDI or anti-Trx mAbs, MDM were infected (in duplicate, in the presence of the inhibitors) for 4 h with the HIV-1 strains indicated, washed twice, and then maintained in MØ medium without the reagents. Cell culture supernatants were harvested and replenished (80% v/v) every three days, and harvested supernatants were stored at −80°C before being analyzed for reverse transcriptase (RT) activity.

We next determined the effect of DTNB on wt infection of human MDM by the laboratory adapted R5 strain, HIV-1_ADA,_ and the minimally passaged isolate, HIV-1_BCF03_, as well as the primary X4 strain, HIV-1_92UG024_. DTNB was able to suppress infection by all three isolates with levels of reverse transcriptase activity approaching those observed for uninfected control MDM, indicating that its effect was not strain specific (Figure 3A,B,C). To identify the disulfide reductases/isomerases involved in HIV-1 infection of MDM and serving as potential targets for DTNB, the effects of specific monoclonal antibodies (mAbs) against PDI and/or Trx, two enzymes previously implicated in HIV-1 Env disulfude bond rearrangements [16-20] were evaluated. The mAbs were applied before and during virus adsorption/fusion; HIV-1 infection was monitored by measuring RT activity at various time points throughout the course of infection. Anti-PDI or anti-Trx mAbs significantly inhibited MDM infection by all three HIV-1 strains tested. However, data obtained from multiple experiments performed with HIV-1_ADA_ established that the anti-Trx mAb was more efficient in reducing RT values and delaying the time of peak RT activity during infection (Figure 3D,E and data not shown), suggesting that Trx may play a greater role in disulfide bond rearrangement in HIV-1 R5 isolates.

### Anti-PDI, but not anti-Trx mAbs, suppressed HIV-1 infection in PM-1 T-cell line

**Figure 4:**
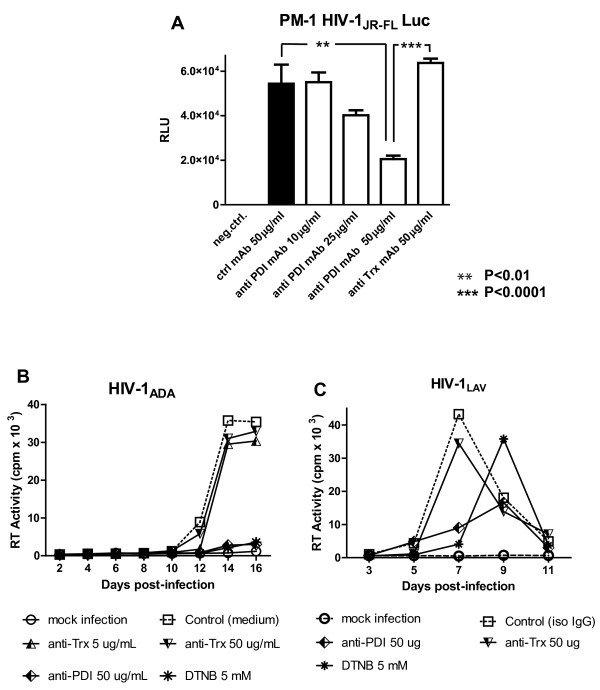
**Anti**-**PDI,but not anti**-**Thioredoxin mAbs inhibit HIV**-**1 infection in PM**-**1 T**-**cell line.A**. PM-1 cells were pre-incubated for 1 h in RPMI-1640 containing the indicated concentrations of nonspecific mouse IgG, anti-PDI or anti-Trx mAbs prior to infection with HIV-1_JR-FL_ Env pseudotyped, Luc reporter gene-encoding virus particles for 2.5 h in the presence of the different mAbs. After the infection period, cells were washed and further incubated for 48 h in RC-10 before being lysed with 0.5% Triton-X100 in PBS and Luc activity was measured. **B**, **C**. Following preincubation for 30 minutes with 5 mM DTNB, anti-PDI or anti-Trx mAbs, PM-1cells were infected with HIV-1_ADA_ (panel **B**) or HIV-1_LAV_ (panel **C**). After infection for 2 h in the presence of the aforementioned inhibitors, the PM-1 cells were washed and further incubated in RC-10 without DTNB or mAbs. Cell culture supernatants were collected every two days and cryopreserved before being evaluated for RT activity.

We next investigated the role of PDI and/or Trx in virus entry and infection of the T cell line, PM-1, which is known to naturally express both CXCR4 and CCR5 and can be infected with R5, as well as X4, HIV-1 strains. We found that anti-PDI mAbs inhibited the infection of PM-1 cells by R5 HIV-1_JR-FL_ Env pseudotyped Luc reporter gene virus particles in a dose dependent manner (Figure 4A). In contrast, the anti-Trx mAbs had no effect on the latter infection, even when used at a maximum concentration of 50 μg/ml. Similar results were obtained when anti-PDI and/or anti-Trx treated PM-1 cells were infected with HIV-1_NL4-3_ Env pseudotyped virions (data not shown). Furthermore, anti-PDI mAb, but not anti-Trx mAb, significantly reduced the RT values and delayed the peak RT activity when PM-1 cells were infected with the R5 laboratory-adapted strain, HIV-1_ADA_, or the X4 strain, HIV-1_LAV_ (Figure 4B,C). Although affinity values for these mAbs could not be obtained from their commercial sources, we established Kd values for the anti-PDI and the anti-Trx mAbs of 15 nM and 8 nM, respectively using surface plasmon resonance (data not shown). The affinities of the two mAbs, as well as the length of incubation (0.5 -1 h pre-incubation plus an additional 4 h during virus adsorption/infection), suggest comparable binding of the two mAbs to their relevant targets, especially when used at the highest concentration of 50 μg/ml (~330nM IgG).

### Cell-type specific requirements for thiol/disulfide exchange during HIV-1 entry and infection in primary human peripheral blood lymphocytes (PBLs) and MDMs

**Figure 5:**
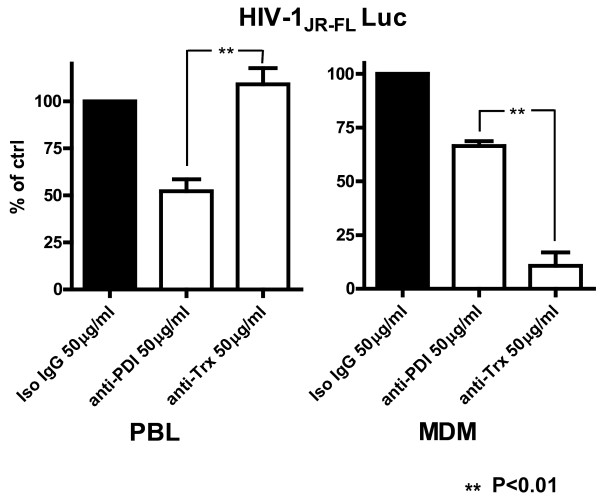
**Cell**-**Type Specific Requirements for Thioredoxin and**/**or PDI in HIV**-**1 Infection in Primary Human Cells.** PHA stimulated PBL were pre-incubated for 1 h with relevant mAbs (50 μg/ml) and then infected for 2.5 h with HIV-1_JR-FL_ Env pseudotyped, Luc reporter gene encoding virus particles in the continued presence of the mAbs. After infection, the cells were washed and further incubated for 48 h in RPMI supplemented with 10% FCS and L-Glutamine prior to lysis (0.5% Triton-X100 in PBS) and Luc activity was then measured. MDM were treated and infected in an identical manner, using DMEM medium. The amount of HIV-1 Env pseudotyped virus particles was adjusted according to the number of cells subjected to infection. Primary human MDM or PBLs from four different donors were prepared and tested as described in Methods. The RLU established for the anti-PDI or anti-Trx mAbs treated cells were calculated as a percent of the mean Luc activity of cells pre-incubated and infected in the presence of nonspecific mouse IgG. The means of the four established values from each donor for the anti-PDI or anti-Trx mAbs treated PBL and MDM are presented in the left and right panel of Figure 5, respectively.

Subsequent to our findings with PM-1 cells, primary human PHA stimulated PBLs were infected with HIV-1_JR-FL_ Env-pseudotyped Luc reporter gene virus particles in the presence and absence of the anti-PDI or anti-Trx mAbs. As expected and consistent with our observations using the PM-1 T cell line, anti-PDI, but not the anti-Trx mAb, reduced the Luc reporter gene activity in primary PBLs isolated from four different donors (Figure 5). Primary MDM were evaluated for the effects of anti-PDI or anti-Trx mAbs on HIV-1_JR-FL_ Env-pseudotyped virus infection using the same experimental system. In contrast to primary human PBLs, the anti-Trx mAb was more effective at inhibiting HIV-1 infection of primary MDM when present during the virus adsorption and entry period. An opposite trend and statistically significant differences (P≤0.01) were established between the anti-PDI and anti-Trx treated cells for the PBL and MDM systems, respectively (Figure 5). Given that the mean Luc activity of the nonspecific IgG treated cells was assumed to be 100%, it was not possible to directly evaluate the statistical significance (P values) of the differences between the control and anti-PDI or control and anti-Trx treated cells in Figure 5. However, the differences between the control and anti-PDI treated PBL and between the control and anti-Trx treated MDM were found to be statistically significant for all donors tested when primary data (RLU) were used to calculate the relevant P values (P≤0.05, not shown).

### PDI and Trx are expressed the cell surface of PM-1 T cells, primary MDM and PBLs

**Figure 6:**
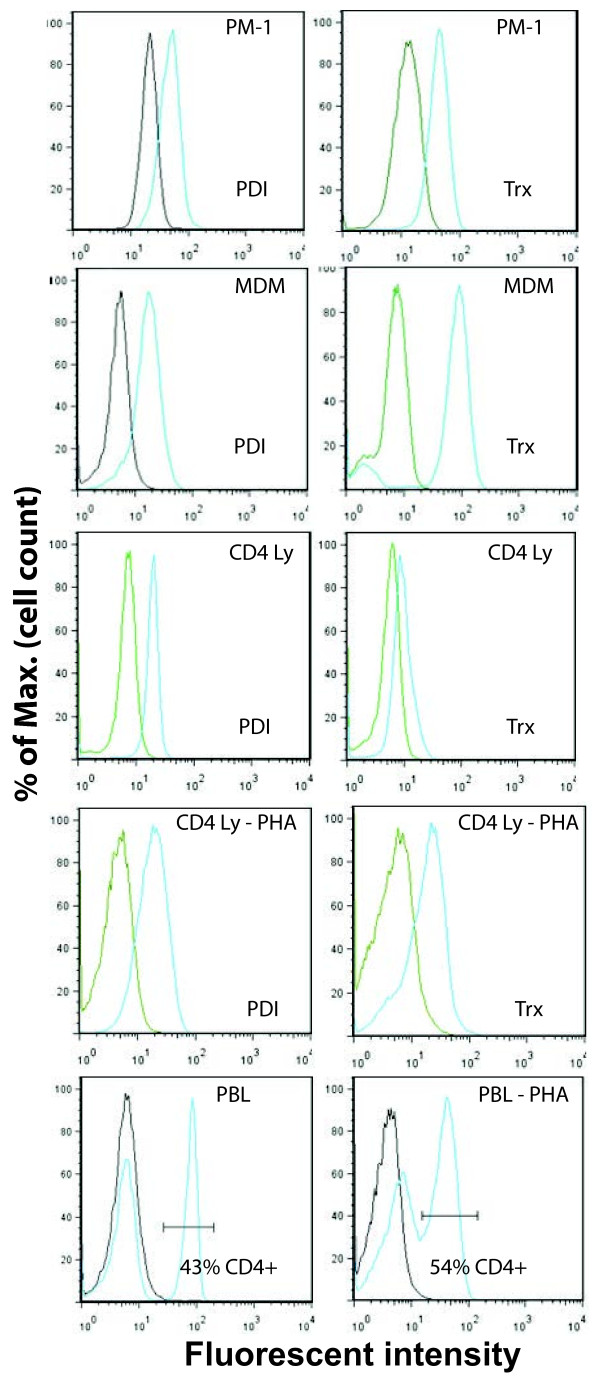
**Cell surface expression of PDI and**/**or Thioredoxin.** PM-1 T cells (panel **A**), primary human MDM (panel **B**) and PBLs (panel **C**) were isolated and cultured as described in Methods. Subsequently, the cells were fixed with 1.6% paraformaldehyde and treated with NH_4_Cl to quench the reactive aldehyde groups before labeling. The PDI and Trx levels on the cell surface were detected by a combination of directly labeled anti-PDI-FITC and anti-Trx-APC mAbs from the same clones used in the HIV-1 infection experiments. In addition, the unstimulated and PHA-P stimulated PBLs were labeled with anti-CD4-Pacific blue conjugated mAb, and the PDI and Trx expression were analyzed on the CD4+ lymphocyte (Ly) population. The results were analyzed and the final graphs were created using FlowJoe software.

In an attempt to delineate the basis for the observed cell-type specific requirements of the thiol/disulfide exchange during virus entry, cell surface expression of PDI and Trx was assessed in the various cell types used for infection. The same mAb clones used to inhibit HIV-1 infection were directly conjugated to different fluorescent dyes (anti-PDI- DyLight 488 and anti-Trx-APC), and used for Flow Cytometry analysis. Although PDI and Trx are predominantly found as intracellular proteins, it is well-documented that these molecules can also be secreted and associate with the plasma membrane [34-36]. Given that cell surface PDI and Trx molecules are peripheral plasma membrane proteins, the cells prepared for Flow Cytometry analysis of cell surface PDI and Trx were washed with DPBS, fixed with 1.6% paraformaldehyde prior to labeling to prevent dissociation from the cell surface and then treated with NH_4_Cl to quench the reactive aldehyde groups before incubation with the specific mAbs as described in Methods. In addition to the anti-PDI and anti-Trx mAbs, PBLs were also incubated with a specific anti-CD4 mAb, directly labeled with the fluorescent marker, Pacific blue, and only those cells that stained positive for CD4 were further analyzed for their PDI and Trx cell surface levels. We found that PM-1 cells and primary MDM expressed both PDI and Trx on their surface (Figure 6A,B). Intriguingly, in contrast to PDI which was present to comparable levels on both unstimulated and PHA-activated CD4+ T cells, Trx expression was very low on unstimulated CD4^+^ lymphocytes, but increased significantly after PHA treatment (Figure 6C). Taken together our findings strongly suggest that the cell type specific effects of anti-PDI and anti-Trx mAbs observed in PM-1 cells, primary MDM, and PHA activated PBLs cannot be attributed to the absence of any enzyme on the plasma membrane of the various cells that have been tested.

## Discussion

There is growing evidence that thiol/disulfide exchange is required for the complex conformational changes that occur during virus-cell fusion. The mature HIV-1 Env is expressed as a trimer of gp120-gp41 heterodimers on the surface of virions and/or infected cells. HIV-1 gp120 consists of five variable domains (V1-V5) interconnected by five more conserved regions (C1-C5), delineated by nine intra-chain disulfide bonds [37,38]. The gp41 ectodomain, in addition to its hydrophobic N-terminus, encompasses two hydrophobic regions with a characteristic coiled-coil structure, designated as heptad repeats 1 and 2, that are connected by a linker sequence forming a conserved disulfide bond [12,39,40]. Although there have been published reports from several independent studies on this topic, the number, location and timing of the HIV-1 Env disulfide bond modifications during virus entry are still not completely elucidated. The majority of disulfide bonds in gp120, delineating or inside domains V1/V2 (Cys^126^-Cys^196^; Cys^119^-Cys^205^; Cys^131^-Cys^157^), V3 (Cys^296^-Cys^331^), V4 (Cys^385^-Cys^418^), C1 (Cys^54^-Cys^74^), C2 (Cys^218^-Cys^247^) and C4 (Cys^378^-Cys^445^), as well as the disulfide bond in gp41, have all been implicated as potential targets for reduction and/or isomerization [11,19,41-43]. There has been some ambiguity with respect to when the disulfide bond rearrangements in gp120 take place: immediately following CD4-gp120 binding [19,20] or after the interaction of gp120 with CD4 and the relevant coreceptor [17,44]. It is reasonable to suggest that some of the disulfide bonds could be modified right after CD4 binding, if such rearrangements facilitate the subsequent coreceptor association while others, such as the disulfide bond straddling the V3 loop and critical for the integrity of the coreceptor binding site [15,45], may undergo modification after the gp120-CD4-coreceptor complex is formed.

It has also been reported that the disulfide bond in the second immunoglobulin-like domain (D2) of CD4 undergoes redox changes necessary for HIV-1 entry but, similar to the situation with HIV-1 Env, the precise timing and role of the CD4 redox modifications during the process of virus fusion remain incompletely characterized [41,46,47]. At present, there is no consensus regarding the particular disulfide reductases/isomerases that may be required for HIV-1 infection. Experimental data from various studies have confirmed the PDI mediated disulfide bond rearrangements in both gp120 [11,20,23,24] and CD4 [26], as well as the suppression of HIV-1 infection by PDI inhibitors [16-20]. However, Trx has also been shown to induce redox changes in gp120 [25,42] and CD4 [25,46] and Ou et al., [25] found that Trx, not PDI, played a major role in HIV-1 Env-mediated fusion. Interestingly, it was recently reported [43] that, despite certain quantitative differences, PDI and Trx generally target the same disulfide bonds within gp120. Auwerx et al., [26] established that another oxidoreductase, Grx1, in addition to PDI, caused a disulfide bond reduction in gp120 and CD4 and also observed a significant (~50%), but incomplete, inhibition of HIV-1_RW009_ infection by polyclonal anti-Grx1 Abs.

Given that there have been only a few reports on the effects of disulfide reductase/isomerase inhibitors on HIV-1 infection of primary human cells [16,19,26], our current study set out to provide a comprehensive investigation of the role of thiol/disulfide exchange in HIV-1 infection using primary human PBMCs, PBLs and MDMs. Using single cycle as well as multi-round replication assays, we initially tested the effects of the non-specific disulfide isomerase/reductase inhibitor, DTNB, employing predominantly R5 strains. In contrast to other viruses, such as hantaviruses [30] where surface glycoproteins express free thiol groups, it is unlikely that DTNB will interact directly with the HIV-1 Env, since those cysteines are interlinked via disulfide bonds. However, if reductase activity is not completely suppressed during the virus-cell fusion process, especially when lower DTNB concentrations are applied, potential alkylation by DTNB of the newly generated thiol groups may contribute to its inhibitory activity. We established that DTNB potently reduced HIV-1 entry and infection in primary cells to levels comparable to the observed inhibition in cell lines (Figure 1 and 2). DTNB also inhibited MDM infection by all strains tested, including the primary, minimally passaged R5 strain BCF03 and X4 strain UG024. Attempts to identify the particular disulfide reductases/isomerases involved in HIV-1 entry by applying specific mAbs, unexpectedly revealed a clearly distinguished specificity among the various cell types tested (Figure 4 and 5). In MDM, anti-Trx mAbs proved to be more potent than the anti-PDI mAbs in suppressing HIV-1 entry and infection. In contrast, these same anti-Trx mAbs had negligible effects on the PM-1 T cell line and primary PBLs, while the anti-PDI mAbs significantly reduced HIV-1 infection in these cells (Figure 3, 4 and 5). Intriguingly, both PDI and Trx were present on the plasma membrane of all cell types tested (Figure 6). Although it appeared that MDMs displayed higher levels of Trx than PDI on their surface, it is unlikely that this fact alone could account for the differences in the HIV-1 inhibitory activities of the anti-Trx and anti-PDI mAbs. In support of this notion, PM-1 cells and PHA stimulated PBLs expressed comparable levels of Trx and PDI on their surface, while anti-PDI and anti-Trx mAbs clearly exerted distinct patterns on HIV-1 infection when compared to each other or to their effects on primary human MDM (Figure 4 and 5). It was previously shown that gp120, CD4, CXCR4 and PDI co-localize on the cell surface [20] and we speculated that a preferential temporal association of gp120, CD4 and the relevant corecepor with either PDI or Trx might be the reason for the observed cell-type specific oxidoreductase involvement in HIV-1 entry. Molecular docking analysis revealed that PDI may interact with the third immunoglobulin-like (D3) region of CD4 [10]. D3 contains one of the glycosylation sites in human CD4 [48] and it is conceivable that subtle differences in CD4 glycosylation may result in its preferential interaction with PDI in some cell types, but not in others. CD4 is also known to interact with the tyrosine kinase p56^lck^ in PBLs and T cell lines, but not in monocytes and MDM [49], which may also influence its behavior on the cell surface in a cell-type dependent manner. CCR5 and CXCR4 heterogeneity [50], as well as their preferential cell type dependent interactions with CD4 [45], may also be contributing factors to the cell-type specific disulfide reductase/isomerase involvement in HIV-1 entry. Alternatively, or in combination with the parameters already discussed, there may be differences in the way T lymphocytes/T cell lines and macrophages regulate the balance between the oxidized and reduced forms of PDI and/or Trx on their surface, which in turn may favor the preferential involvement of one of these enzymes during HIV-1 entry. Cell surface molecules other than CD4 and CCR5 may also interact with PDI or Trx and influence their redox status and/or availability during HIV-1 entry. It was recently reported [51] that galectin-9 binds to and increases the retention of PDI on the cell surface, thus facilitating Th2 lymphocyte migration and HIV-1 infection most likely through modulation of the cell surface redox environment.

There is evidence that, in addition to PDI and Trx, there may be other disulfide reductases like Grx1 [26] participating in the HIV-1 Env-mediated fusion. Whether these alternative disulfide reductases/isomerases display cell-type specific activities remains to be established. Interestingly, both PDI (Figure 5) and Grx1 [26] significantly, but incompletely (~50%) reduced HIV-1 infection in PBLs and/or PBMCs, suggesting that certain oxidoreductases may substitute for each other during HIV-1 entry.

Finally, we looked at our results from the perspective of HIV-1 infection *in vivo* in an attempt to better understand HIV-1 pathogenesis. The redox imbalance is a hallmark of HIV-1 infection, resulting in a complex chain of events at intra- and extracellular levels that influence HIV-1 replication (reviewed in [52,53]). The finding that freshly isolated PBLs, in contrast to PHA-stimulated cells, express ample amounts of PDI yet express relatively low levels of Trx on their surface, may provide insights regarding the predominant utilization of PDI by CD4^+^ lymphocytes, the entry of HIV-1 in resting T cells, and the establishment of virus reservoirs. Given that MDM utilize predominantly Trx during HIV-1 entry (Figures 3D,E and 5), the observed elevation of plasma Trx during chronic stages of HIV disease [54,55] may enhance macrophage infection and help sustain high viremia even when very low levels of CD4^+^ T-cells exist. In this regard, plasma Trx levels showed a significant inverse correlation with the survival rate of HIV-1-infected patients having a CD4^+^ T cell count below 200 cells/μL [55].

The enormous potential of HIV-1 to mutate [56], its ability to establish long-lasting viral reservoirs that are currently impossible to completely eradicate even with the highly active anti-retroviral therapy (HAART) [57], along with the emergence of resistant strains to the CCR5 antagonist maraviroc (Selzentry) (reviewed in [58]) and/or the fusion inhibitor T-20 (Enfuvirtide) [59], emphasize the importance of developing new and effective therapies to suppress virus entry. Therefore, the identification of more conserved host cellular factors, such as the disulfide reductases/ isomerases involved in HIV-1 pathogenesis, and our increased understanding of their mechanisms of action, may facilitate the future development of new antiviral modalities.

## Conclusions

Our findings clearly establish the role of thiol/disulfide exchange in HIV-1 entry and infection of primary T cells and MDM by a variety of strains, including isolates that have been minimally passaged. Regarding the involvement of particular disulfide isomerases/reductases in this process, our results also demonstrate a cell-type dependent specificity and may provide an explanation for differences among previously published studies. From an *in vivo* perspective, the preferential utilization of PDI may be relevant to the HIV-1 entry and establishment of virus reservoirs in resting CD4^+^ cells, while the elevated levels of Trx reported in the chronic stages of HIV-1 infection may facilitate the virus entry in macrophages and help to sustain high viremia during the decline of T lymphocytes.

## Methods

### Reagents

5,5`-dithiobis[2-nitrobenzoic acid] (DTNB) was purchased from Sigma-Aldrich (St. Louis, MO). Fetal bovine serum (FBS) was obtained from Hyclone (Logan, UT). Phosphate Buffered Saline (PBS) and RPMI 1640 medium were purchased from Lonza (Walkersville, MD). Phenol red-free Dulbecco’s Modified Eagle’s Medium (DMEM), HEPES buffer solution (1 M) and the CCF2/AM β-lactamase Loading Kit (GeneBLAzer Reporter Assay) were purchased from Invitrogen Corp. (Carlsbad, CA). Penicillin/Streptomycin antibiotic solution (10,000U/ml and 10,000 μg/ml, respectively), sodium pyruvate (100 mM), Dulbecco’s Phosphate Buffered Saline (DPBS) and L-glutamine (200 mM) were obtained from Lonza (Walkersville, MD). The Luciferase Assay System and the FuGENE 6 transfection reagent were purchased from Promega Corp. (Madison, WI) and Roche Applied Science (Indianapolis, IN), respectively.

The HIV-1_ADA_ virus stock was purchased from Advanced Biotechnologies (Columbia, MD). HIV-1_BCF03_[60] and HIV-1_92UG024_ (the UNAIDS Network for HIV Isolation and Characterization and DAIDS, NIAID) were received through the AIDS Research and Reference Reagent Program, NIAID, NIH.

The plasmids pNL4-3 e-R+ Luc, pSV7d-JR-FLgp160, pSV7d-AD8gp160 and pMM310 (pcDNA3.1(Zeo)-Vpr-BlaM) were provided by Dr. R. Doms (Univ. Penn.), Dr. G. Quinnan (USUHS, Bethesda, MD), and Dr. M. Miller (Merck & Co., Inc., West Point, PA), respectively.

Mouse monoclonal anti-PDI antibody (mAb), clone 1D3, and polyclonal rabbit anti-PDI antibody (pAb) were purchased from Enzo Life Sciences (formerly Stressgen and Assay Designs) (Ann Arbor, MI). The mouse anti-human Trx mAb, clone 2B1, was obtained from AbD Serotec (Raleigh, NC). Unlabeled, Pacific Blue, FITC or PE-Cy5 conjugated isotype IgG controls, anti-human CD4 (clone Leu3a), anti-human CCR5 (clone CTC5), anti-human CXCR-4 (clone 12 G5) and goat anti-mouse F(ab)_2_–AlexaFluor 488 were purchased from eBiosciences (San Diego, CA), BD Biosciences (San Jose, CA) and Life Technologies (Grand Island, NY). Human FcR blocking reagent was purchased from Miltenyi Biotec (Auburn, CA) and the LYNX Rapid APC Antibody Conjugation kit was obtained fron AbD Serotec.

### Cells and cell culture conditions

The 293 T, JC53 [61], HOS CD4^+^/CCR5^+^ and PM-1 cell lines were obtained from Dr. G. Quinnan (USUHS, Bethesda, MD), Dr. D. Dimitrov (NCI, Frederick), the AIDS Research and Reference Reagent Program, NIAID, NIH: Dr. M. Reitz [62], and the American Type Culture Collection (ATCC), respectively. The cell lines were maintained in DMEM (293 T, JC53 and HOS) or RPMI-1640 (PM-1), supplemented with 10% FCS, 2 mM L-glutamine, and antibiotics (DM-10 or RC-10) at 37°C in a humidified, 5% CO_2_ atmosphere. Human PBMC were isolated by Ficoll-Paque^TM^ gradient centrifugation from leukapheresis of healthy, human donors seronegative for HIV-1, HIV-2, hepatitis B and hepatitis C. Monocytes and lymphocytes were further separated using countercurrent centrifugal cell elutriation as previously described [63]. For the Luc reporter gene and the BlaM entry assays, macrophages were prepared from elutriated monocytes by differentiation in 100 mm square Petri dishes (Bibbi Sterilin Ltd., Stone Staffs, UK) in DMEM supplemented with 10% human serum pooled from multiple normal donors, 2 mM L-glutamine and antibiotics (MØ medium) [64,65]. Human MDM were obtained after 7 to 14 days of differentiation in the absence of exogenous growth factors and were either used immediately after the differentiation period or kept frozen in liquid nitrogen. The day before the experiment, frozen cells were thawed, washed, centrifuged, and resuspended in DM-10 medium prior to incubation at 37°C overnight. For the wild type (wt) HIV-1 infection experiments, monocytes were differentiated for 7 days in 6-well tissue culture plates (Costar; MA), harvested and re-plated into 24-well plates (Nunc, Roskilde, Denmark) 24 to 48 h prior to HIV-1 infection (0.75 x 10^6^ cells/well in 1.5 mL of medium). PBL were either kept frozen or used immediately after elutriation. After stimulation for 72 h with PHA-P 10 μg/ml (Sigma-Aldrich, St. Louis, MO), PBL were washed, counted and infected with HIV-1 Env pseudotyped or HIV-1 BlaM-containing virions in 24 well plates (10^6^ cells/well).

### Surface plasmon resonance analysis

The experiments were performed using a Biacore T200 system (GE Healthcare). The two mAbs tested were immobilized on CM5 sensors either directly (anti-Trx) or indirectly (anti-PDI) via anti-mouse Fc rabbit IgG (Jackson Immunoresearch Laboratories, West Grove, PA). Both the anti-Trx mAb and the rabbit anti-mouse Fc IgG were cross-linked to the CM5 sensors using an amine coupling kit (GE Healthcare). Human recombinant Trx and PDI were obtained from Sigma-Aldrich (St. Louis, MO) and Enzo Life Sciences (Farmingdale, NY), respectively, and the binding to their relevant mAbs was tested over a concentration range of 12.5 to 400 nM. After each cycle, the surfaces of the CM5 sensors were regenerated by applying low pH glycine buffer, as the efficiency of the regeneration procedures were verified in prior pilot experiments. The generated data were evaluated using the Biacore T200 software for kinetic analysis.

### Flow cytometry

CD4 and PDI were detected using the directly conjugated mAbs anti-CD4 (clone Leu3a)-Pacific Blue (BD Pharmingen) and anti-PDI (clone 1D3)-DyLight 488 (Assay Designs), respectively. Relevant isotype controls were obtained from the same companies. The anti-Trx mAb, clone 2B1, (AbD Serotec) and relevant isotype control IgG were labeled with the fluorescent dye APC using the LYNX Rapid APC Antibody Conjugation kit, following the manufacturer’s instructions (AbD Serotec). The non-overlapping emission and excitation spectra of Pacific blue, DyLight 488 and APC allowed simultaneous labeling with the three different mAbs and subsequent Flow Cytometry analysis with minimal interference. The PM-1 cells and the primary human lymphocytes were washed with DPBS (x1), fixed in 1.6% paraformaldehyde, washed again with DPBS (x1) and incubated for 30 min in 10 mM NH_4_Cl to quench the free aldehyde groups and reduce unspecific antibody binding. Primary MDM were treated identically after detachment from plastic by washing and incubation with DPBS (Ca^2+^ and Mg^2+^ free) at 4°C, followed by pipetting and gentle scraping, if necessary. Cells were further washed (x1) in DPBS and incubated overnight in DPBS plus 3% bovine serum albumin (BSA). Subsequently, the cells were labeled simultaneously with the anti-CD4, anti-PDI, and anti-Trx mAbs or the relevant isotype controls (1 h at 4°C in DPBS, 3% BSA, 2.5 μg of each of the mAbs per 10^6^ cells), washed 3 times with DPBS and analyzed using the LSR II BD Cell Analyzer. For detection of CCR5 and/or CXCR4, cells were initially labeled with the relevant antibodies on ice and subsequently fixed in 1.6% paraformaldehyde before Flow Cytometry analysis. CCR5 and CXR4 were detected either by using PE-Cy5 conjugated specific antibodies (BD Biosciences) or using non-conjugated anti-CXCR4 (clone 12 G5) and anti-CCR5 (clone 2D7) mAbs, followed by goat anti-mouse FITC-conjugated IgG F_(ab)2,_ (Life Technologies, NY).

### Infection studies using HIV-1 Env pseudotyped virus particles

For the reporter gene HIV-1-Env pseudotyping system [66], viral stocks were prepared by transfecting 293 T cells with plasmids encoding the Luc virus backbone pNL4-3-Luc e^-^R^+^ and pSV7d-JR-FLgp160 or pSV7d-AD8gp160. After transfection, the cells were washed extensively with DMEM and further incubated for 24–48 h. The resulting supernatants were clarified by centrifugation for 10 min at 1500 rpm, filtered through low protein binding 45 μM syringe filters (Millipore, Bedford, MA) and used immediately or kept at 4°C for up to 48 h. CD4/coreceptor positive cells were prepared in 96 (50x10^3^ cells/well) or 48 well (1.5x10^5^ cells/well) plates and infected with 25 or 50 μl virus suspension per well, respectively. No DEAE-Dextran or polybrene were used to facilitate fusion. DTNB, anti-PDI or anti-Trx mAbs (in serum free DMEM) were applied as indicated in the figure legends. After the 2.5 h period for virus infection (in a serum free medium), the cells were extensively washed and then incubated for 48 h (in medium supplemented with 10% FBS) prior to lysis with 0.5% Triton-100 in PBS (Luc reporter gene studies). A 50 μl aliquot of the resulting lysate was assayed for Luc activity using the appropriate substrate (Promega, Madison, WI). To exclude the possibility of DTNB having an effect on Luc expression at a post-entry level, HOS cells were electroporated with the pNL4-3 e-R+ plasmid, incubated for 3.5 h on the next day in concentrations of DTNB used in the virus infection experiments and lysed and tested for Luc activity 48 h later. The statistical analysis of the experiments employing HIV-1 Env pseudotyped, Luc reporter gene encoding virus particles was performed using the GraphPad Prism scientific software (t-test).

### HIV-1 entry assay

BlaM-containing virus particles were produced by co-transfection of 293 T cells with a plasmid encoding HIV-1_AD8_ proviral DNA and pMM310. The pMM310 construct encodes BlaM fused to the amino-terminus of the viral protein Vpr, which directs BlaM into the forming virions [33,67]. After pre-incubation and infection for 2.5 h (in a serum free medium), the cells were extensively washed and loaded with the fluorescent dye CCF2/AM (2 μM final concentration) for 1 h, washed to remove the extracellular dye and incubated in phenol red free DMEM plus 10% FCS, 2 mM L-Glutamine and 25 mM HEPES in the presence of the nonspecific anion transport inhibitor, probenecid (2.5 mM), for 12–14 h prior to fixing with 1.6% paraformaldehyde [32]. The extent of CCF2/AM cleavage by the virus-introduced intracellular BlaM, detected by the change in dye emission from the green to the blue spectrum, was evaluated by measuring the fluorescence using Laser Scanning Cytometer (CompuCyte, Cambridge, MA) or BD LSR II Cell Analyzer generating light at 407 nm and equipped with HQ460/30 and HQ530/20 filters for detection of the blue and green emission, respectively. Individual negative controls (mock infection) were prepared for both medium- or DTNB-treated cells to define more precisely the region of BlaM positive cells in the Laser Scanning or Flow Cytometry experiments. To exclude the possibility of DTNB having an effect on BlaM activity, HOS and/or JC53 cells were electroporated with the parental (pCDNA3.1) or the Vpr-BlaM encoding plasmid, incubated for 3.5 h on the next day in 5 mM DTNB, washed (x2), loaded with fluorescent dye CCF2/AM and analyzed by Flow Cytometry as described for the virus entry experiments.

### HIV-1 infection of human MDM and PM-1 cells

24–48 h after being seeded in a 24 well plate, the macrophages were washed, incubated 30 min with different disulfide reductase/isomerase inhibitors or the appropriate controls, and infected for 4 h with select HIV-1 strains in the presence of the reagents. Subsequently, the cells were washed and further incubated in MØ medium without disulfide reductase inhibitors. The progression of virus replication was monitored by measuring reverse transcriptase (RT) activity as follows: every 3^rd^ day, ~80% of the culture medium was harvested and replaced, with the collected medium stored at −80°C until evaluation.

Following preincubation for 30 minutes with 5 mM DTNB, anti-PDI or anti-Trx mAbs, PM-1 cells were infected with HIV-1_ADA_ (panel B) or HIV-1_LAV_ (panel C) in 24 well plates. After infection for 2 h in the presence of the aforementioned inhibitors, the PM-1 cells were washed and further incubated in RC-10 without DTNB or mAbs. Cell culture supernatants were collected every two days and cryopreserved before being evaluated for RT activity. The RT assay employed in the current study was a ^3^H-based modification of the methods described by Hoffman et al. [68]. For both MDM and PM-1 cells the values shown reflect the average of duplicate samples (cpm/25 μl) that differed by not more than 15%.

## Competing interests

The authors declare that they have no competing interests.

## Authors’ contributions

TS and MP designed and conducted the experiments, analyzed the data and wrote the manuscript (equal contribution). CL and FS performed experiments and analyzed data. KC and CB coordinated the study, analyzed data and have been involved in writing the manuscript. All authors have read and approved the final manuscript.

## References

[B1] Abell BA Brown DT Sindbis virus membrane fusion is mediated by reduction of glycoprotein disulfide bridges at the cell surface J Virol 1993 67 9 5496 5501 8350409 10.1128/jvi.67.9.5496-5501.1993 PMC237952

[B2] Markovic I Pulyaeva H Sokoloff A Chernomordik LV Membrane fusion mediated by baculovirus gp64 involves assembly of stable gp64 trimers into multiprotein aggregates J Cell Biol 1998 143 5 1155 1166 10.1083/jcb.143.5.1155 9832546 PMC2133075

[B3] Locker JK Griffiths G An unconventional role for cytoplasmic disulfide bonds in vaccinia virus proteins J Cell Biol 1999 144 2 267 279 10.1083/jcb.144.2.267 9922453 PMC2132897

[B4] Wieringa R de Vries AA Rottier PJ Formation of disulfide-linked complexes between the three minor envelope glycoproteins (GP2b, GP3, and GP4) of equine arteritis virus J Virol 2003 77 11 6216 6226 10.1128/JVI.77.11.6216-6226.2003 12743278 PMC155002

[B5] Wallin M Loving R Ekstrom M Li K Garoff H Kinetic analyses of the surface-transmembrane disulfide bond isomerization-controlled fusion activation pathway in Moloney murine leukemia virus J Virol 2005 79 22 13856 13864 10.1128/JVI.79.22.13856-13864.2005 16254321 PMC1280236

[B6] Jain S McGinnes LW Morrison TG Thiol/disulfide exchange is required for membrane fusion directed by the Newcastle disease virus fusion protein J Virol 2007 81 5 2328 2339 10.1128/JVI.01940-06 17151113 PMC1865930

[B7] Jain S McGinnes LW Morrison TG Role of thiol/disulfide exchange in newcastle disease virus entry J Virol 2009 83 1 241 249 10.1128/JVI.01407-08 18922867 PMC2612340

[B8] Mahon PJ Mirza AM Musich TA Iorio RM Engineered intermonomeric disulfide bonds in the globular domain of Newcastle disease virus hemagglutinin-neuraminidase protein: implications for the mechanism of fusion promotion J Virol 2008 82 21 10386 10396 10.1128/JVI.00581-08 18753211 PMC2573173

[B9] Abou-Jaoude G Sureau C Entry of hepatitis delta virus requires the conserved cysteine residues of the hepatitis B virus envelope protein antigenic loop and is blocked by inhibitors of thiol-disulfide exchange J Virol 2007 81 23 13057 13066 10.1128/JVI.01495-07 17898062 PMC2169099

[B10] Ryser HJ Fluckiger R Progress in targeting HIV-1 entry Drug Discov Today 2005 10 16 1085 1094 10.1016/S1359-6446(05)03550-6 16182193

[B11] Fenouillet E Barbouche R Jones IM Cell entry by enveloped viruses: redox considerations for HIV and SARS-coronavirus Antioxid Redox Signal 2007 9 8 1009 1034 10.1089/ars.2007.1639 17567241

[B12] Berger EA Murphy PM Farber JM Chemokine receptors as HIV-1 coreceptors: roles in viral entry, tropism, and disease Annu Rev Immunol 1999 17 657 700 10.1146/annurev.immunol.17.1.657 10358771

[B13] Doms RW Moore JP HIV-1 membrane fusion: targets of opportunity J Cell Biol 2000 151 2 F9 F14 10.1083/jcb.151.2.F9 11038194 PMC2192632

[B14] Eckert DM Kim PS Mechanisms of viral membrane fusion and its inhibition Annu Rev Biochem 2001 70 777 810 10.1146/annurev.biochem.70.1.777 11395423

[B15] van Anken E Sanders RW Liscaljet IM Land A Bontjer I Tillemans S Nabatov AA Paxton WA Berkhout B Braakman I Only five of 10 strictly conserved disulfide bonds are essential for folding and eight for function of the HIV-1 envelope glycoprotein Mol Biol Cell 2008 19 10 4298 4309 10.1091/mbc.E07-12-1282 18653472 PMC2555952

[B16] Ryser HJ Levy EM Mandel R DiSciullo GJ Inhibition of human immunodeficiency virus infection by agents that interfere with thiol-disulfide interchange upon virus-receptor interaction Proc Natl Acad Sci U S A 1994 91 10 4559 4563 10.1073/pnas.91.10.4559 8183947 PMC43825

[B17] Barbouche R Miquelis R Jones IM Fenouillet E Protein-disulfide isomerase-mediated reduction of two disulfide bonds of HIV envelope glycoprotein 120 occurs post-CXCR4 binding and is required for fusion J Biol Chem 2003 278 5 3131 3136 10.1074/jbc.M205467200 12218052

[B18] Fenouillet E Barbouche R Courageot J Miquelis R The catalytic activity of protein disulfide isomerase is involved in human immunodeficiency virus envelope-mediated membrane fusion after CD4 cell binding J Infect Dis 2001 183 5 744 752 10.1086/318823 11181151

[B19] Gallina A Hanley TM Mandel R Trahey M Broder CC Viglianti GA Ryser HJ Inhibitors of protein-disulfide isomerase prevent cleavage of disulfide bonds in receptor-bound glycoprotein 120 and prevent HIV-1 entry J Biol Chem 2002 277 52 50579 50588 10.1074/jbc.M204547200 12218051

[B20] Markovic I Stantchev TS Fields KH Tiffany LJ Tomic M Weiss CD Broder CC Strebel K Clouse KA Thiol/disulfide exchange is a prerequisite for CXCR4-tropic HIV-1 envelope-mediated T-cell fusion during viral entry Blood 2004 103 5 1586 1594 10.1182/blood-2003-05-1390 14592831

[B21] Khan MM Simizu S Lai NS Kawatani M Shimizu T Osada H Discovery of a small molecule PDI inhibitor that inhibits reduction of HIV-1 envelope glycoprotein gp120 ACS Chem Biol 2011 6 3 245 251 10.1021/cb100387r 21121641

[B22] Lara HH Ixtepan-Turrent L Garza-Trevino EN Flores-Tevino SM Borkow G Rodriguez-Padilla C Antiviral propierties of 5,5'-dithiobis-2-nitrobenzoic acid and bacitracin against T-tropic human immunodeficiency virus type 1 Virol J 2011 8 137 10.1186/1743-422X-8-137 21435237 PMC3078101

[B23] Wang Z Zhou Z Guo ZY Chi CW Snapshot of the interaction between HIV envelope glycoprotein 120 and protein disulfide isomerase Acta Biochim Biophys Sin (Shanghai) 2010 42 5 358 362 10.1093/abbs/gmq024 20458450

[B24] Papandreou MJ Barbouche R Guieu R Rivera S Fantini J Khrestchatisky M Jones IM Fenouillet E Mapping of domains on HIV envelope protein mediating association with calnexin and protein-disulfide isomerase J Biol Chem 2010 285 18 13788 13796 10.1074/jbc.M109.066670 20202930 PMC2859542

[B25] Ou W Silver J Role of protein disulfide isomerase and other thiol-reactive proteins in HIV-1 envelope protein-mediated fusion Virology 2006 350 2 406 417 10.1016/j.virol.2006.01.041 16507315

[B26] Auwerx J Isacsson O Soderlund J Balzarini J Johansson M Lundberg M Human glutaredoxin-1 catalyzes the reduction of HIV-1 gp120 and CD4 disulfides and its inhibition reduces HIV-1 replication Int J Biochem Cell Biol 2009 41 6 1269 1275 10.1016/j.biocel.2008.10.031 19038358

[B27] Moore JP Kitchen SG Pugach P Zack JA The CCR5 and CXCR4 coreceptors–central to understanding the transmission and pathogenesis of human immunodeficiency virus type 1 infection AIDS Res Hum Retroviruses 2004 20 1 111 126 10.1089/088922204322749567 15000703

[B28] Yi Y Isaacs SN Williams DA Frank I Schols D De Clercq E Kolson DL Collman RG Role of CXCR4 in cell-cell fusion and infection of monocyte-derived macrophages by primary human immunodeficiency virus type 1 (HIV-1) strains: two distinct mechanisms of HIV-1 dual tropism J Virol 1999 73 9 7117 7125 10438797 10.1128/jvi.73.9.7117-7125.1999 PMC104231

[B29] Mongin AA Nedvetsky PI Fedorovich SV Depolarization of isolated brain nerve endings by nitric oxide donors: membrane mechanisms Biochemistry (Mosc) 1998 63 6 662 670 9668206

[B30] Strandin T Hepojoki J Wang H Vaheri A Lankinen H Inactivation of hantaviruses by N-ethylmaleimide preserves virion integrity J Gen Virol 2010 92 Pt 5 1189 1198 21289161 10.1099/vir.0.027896-0

[B31] Cohen O Weissman D Fauci AS Paul WE The immunopathogenesis of HIV infection Fundamental Immunology 1998 Lippincott-Raven, Philadelphia 1511 1534

[B32] Cavrois M De Noronha C Greene WC A sensitive and specific enzyme-based assay detecting HIV-1 virion fusion in primary T lymphocytes Nat Biotechnol 2002 20 11 1151 1154 10.1038/nbt745 12355096

[B33] Tobiume M Lineberger JE Lundquist CA Miller MD Aiken C Nef does not affect the efficiency of human immunodeficiency virus type 1 fusion with target cells J Virol 2003 77 19 10645 10650 10.1128/JVI.77.19.10645-10650.2003 12970449 PMC228506

[B34] Arner ES Holmgren A Physiological functions of thioredoxin and thioredoxin reductase Eur J Biochem 2000 267 20 6102 6109 10.1046/j.1432-1327.2000.01701.x 11012661

[B35] Turano C Coppari S Altieri F Ferraro A Proteins of the PDI family: unpredicted non-ER locations and functions J Cell Physiol 2002 193 2 154 163 10.1002/jcp.10172 12384992

[B36] Wilkinson B Gilbert HF Protein disulfide isomerase Biochim Biophys Acta 2004 1699 1–2 35 44 15158710 10.1016/j.bbapap.2004.02.017

[B37] Leonard CK Spellman MW Riddle L Harris RJ Thomas JN Gregory TJ Assignment of intrachain disulfide bonds and characterization of potential glycosylation sites of the type 1 recombinant human immunodeficiency virus envelope glycoprotein (gp120) expressed in Chinese hamster ovary cells J Biol Chem 1990 265 18 10373 10382 2355006

[B38] Hunter E Coffin SH, Hughes SH, Varmus HE Viral entry and receptors Retroviruses 1997 Cold Spring Harbor Laboratory Press, New York 71 119 21433347

[B39] Gallaher WR Ball JM Garry RF Griffin MC Montelaro RC A general model for the transmembrane proteins of HIV and other retroviruses AIDS Res Hum Retroviruses 1989 5 4 431 440 10.1089/aid.1989.5.431 2788443

[B40] Chan DC Chutkowski CT Kim PS Evidence that a prominent cavity in the coiled coil of HIV type 1 gp41 is an attractive drug target Proc Natl Acad Sci U S A 1998 95 26 15613 15617 10.1073/pnas.95.26.15613 9861018 PMC28092

[B41] Matthias LJ Hogg PJ Redox control on the cell surface: implications for HIV-1 entry Antioxid Redox Signal 2003 5 1 133 138 10.1089/152308603321223621 12626125

[B42] Azimi I Matthias LJ Center RJ Wong JW Hogg PJ Disulfide bond that constrains the HIV-1 gp120 V3 domain is cleaved by thioredoxin J Biol Chem 2010 285 51 40072 40080 10.1074/jbc.M110.185371 20943653 PMC3000989

[B43] Reiser K Francois KO Schols D Bergman T Jornvall H Balzarini J Karlsson A Lundberg M Thioredoxin-1 and protein disulfide isomerase catalyze the reduction of similar disulfides in HIV gp120 Int J Biochem Cell Biol 2012 44 3 556 562 10.1016/j.biocel.2011.12.015 22230366

[B44] Billington J Hickling TP Munro GH Halai C Chung R Dodson GG Daniels RS Stability of a receptor-binding active human immunodeficiency virus type 1 recombinant gp140 trimer conferred by intermonomer disulfide bonding of the V3 loop: differential effects of protein disulfide isomerase on CD4 and coreceptor binding J Virol 2007 81 9 4604 4614 10.1128/JVI.02138-06 17301129 PMC1900172

[B45] Huang CC Tang M Zhang MY Majeed S Montabana E Stanfield RL Dimitrov DS Korber B Sodroski J Wilson IA Structure of a V3-containing HIV-1 gp120 core Science 2005 310 5750 1025 1028 10.1126/science.1118398 16284180 PMC2408531

[B46] Matthias LJ Yam PT Jiang XM Vandegraaff N Li P Poumbourios P Donoghue N Hogg PJ Disulfide exchange in domain 2 of CD4 is required for entry of HIV-1 Nat Immunol 2002 3 8 727 732 12089508 10.1038/ni815

[B47] Goldsmith MA Doms RW HIV entry: are all receptors created equal? Nat Immunol 2002 3 8 709 710 12089511 10.1038/ni819

[B48] Brady RL Barclay AN The structure of CD4 Curr Top Microbiol Immunol 1996 205 1 18 10.1007/978-3-642-79798-9_1 8575192

[B49] Graziani-Bowering G Filion LG Thibault P Kozlowski M CD4 is active as a signaling molecule on the human monocytic cell line Thp-1 Exp Cell Res 2002 279 1 141 152 10.1006/excr.2002.5581 12213222

[B50] Lapham CK Zaitseva MB Lee S Romanstseva T Golding H Fusion of monocytes and macrophages with HIV-1 correlates with biochemical properties of CXCR4 and CCR5 Nat Med 1999 5 3 303 308 10.1038/6523 10086386

[B51] Bi S Hong PW Lee B Baum LG Galectin-9 binding to cell surface protein disulfide isomerase regulates the redox environment to enhance T-cell migration and HIV entry Proc Natl Acad Sci U S A 2011 108 26 10650 10655 10.1073/pnas.1017954108 21670307 PMC3127870

[B52] Nakamura H Masutani H Yodoi J Redox imbalance and its control in HIV infection Antioxid Redox Signal 2002 4 3 455 464 10.1089/15230860260196245 12215212

[B53] Masutani H Ueda S Yodoi J The thioredoxin system in retroviral infection and apoptosis Cell Death Differ 2005 12 Suppl 1 991 998 15818395 10.1038/sj.cdd.4401625

[B54] Nakamura H De Rosa S Roederer M Anderson MT Dubs JG Yodoi J Holmgren A Herzenberg LA Herzenberg LA Elevation of plasma thioredoxin levels in HIV-infected individuals Int Immunol 1996 8 4 603 611 10.1093/intimm/8.4.603 8671648

[B55] Nakamura H De Rosa SC Yodoi J Holmgren A Ghezzi P Herzenberg LA Herzenberg LA Chronic elevation of plasma thioredoxin: inhibition of chemotaxis and curtailment of life expectancy in AIDS Proc Natl Acad Sci U S A 2001 98 5 2688 2693 10.1073/pnas.041624998 11226300 PMC30199

[B56] Sarafianos SG Marchand B Das K Himmel DM Parniak MA Hughes SH Arnold E Structure and function of HIV-1 reverse transcriptase: molecular mechanisms of polymerization and inhibition J Mol Biol 2009 385 3 693 713 10.1016/j.jmb.2008.10.071 19022262 PMC2881421

[B57] Tyagi M Bukrinsky M HIV latency: the major hurdle in HIV eradication Mol Med 2012 18 1 1096 1108 22692576 10.2119/molmed.2012.00194 PMC3475336

[B58] Moore JP Kuritzkes DR A piece de resistance: how HIV-1 escapes small molecule CCR5 inhibitors Curr Opin HIV AIDS 2009 4 2 118 124 10.1097/COH.0b013e3283223d46 19339950 PMC2896203

[B59] Miller MD Hazuda DJ HIV resistance to the fusion inhibitor enfuvirtide: mechanisms and clinical implications Drug Resist Updat 2004 7 2 89 95 10.1016/j.drup.2004.03.003 15158765

[B60] Loussert-Ajaka I Chaix ML Korber B Letourneur F Gomas E Allen E Ly TD Brun-Vezinet F Simon F Saragosti S Variability of human immunodeficiency virus type 1 group O strains isolated from Cameroonian patients living in France J Virol 1995 69 9 5640 5649 7637010 10.1128/jvi.69.9.5640-5649.1995 PMC189421

[B61] Platt EJ Wehrly K Kuhmann SE Chesebro B Kabat D Effects of CCR5 and CD4 cell surface concentrations on infections by macrophagetropic isolates of human immunodeficiency virus type 1 J Virol 1998 72 4 2855 2864 9525605 10.1128/jvi.72.4.2855-2864.1998 PMC109730

[B62] Lusso P Cocchi F Balotta C Markham PD Louie A Farci P Pal R Gallo RC Reitz MS Jr Growth of macrophage-tropic and primary human immunodeficiency virus type 1 (HIV-1) isolates in a unique CD4+ T-cell clone (PM1): failure to downregulate CD4 and to interfere with cell-line-tropic HIV-1 J Virol 1995 69 6 3712 3720 7745720 10.1128/jvi.69.6.3712-3720.1995 PMC189087

[B63] Gerrard TL Jurgensen CH Fauci AS Differential effect of monoclonal anti-DR antibody on monocytes in antigen- and mitogen-stimulated responses: mechanism of inhibition and relationship to interleukin 1 secretion Cell Immunol 1983 82 2 394 402 10.1016/0008-8749(83)90172-7 6606492

[B64] Lazdins JK Woods-Cook K Walker M Alteri E The lipophilic muramyl peptide MTP-PE is a potent inhibitor of HIV replication in macrophages AIDS Res Hum Retroviruses 1990 6 10 1157 1161 10.1089/aid.1990.6.1157 1701314

[B65] Broder CC Kennedy PE Michaels F Berger EA Expression of foreign genes in cultured human primary macrophages using recombinant vaccinia virus vectors Gene 1994 142 2 167 174 10.1016/0378-1119(94)90257-7 8194748

[B66] Connor RI Chen BK Choe S Landau NR Vpr is required for efficient replication of human immunodeficiency virus type-1 in mononuclear phagocytes Virology 1995 206 2 935 944 10.1006/viro.1995.1016 7531918

[B67] Wyma DJ Jiang J Shi J Zhou J Lineberger JE Miller MD Aiken C Coupling of human immunodeficiency virus type 1 fusion to virion maturation: a novel role of the gp41 cytoplasmic tail J Virol 2004 78 7 3429 3435 10.1128/JVI.78.7.3429-3435.2004 15016865 PMC371074

[B68] Hoffman AD Banapour B Levy JA Characterization of the AIDS-associated retrovirus reverse transcriptase and optimal conditions for its detection in virions Virology 1985 147 2 326 335 10.1016/0042-6822(85)90135-7 2416116

